# Polycystic ovary syndrome: a complex condition with psychological, reproductive and metabolic manifestations that impacts on health across the lifespan

**DOI:** 10.1186/1741-7015-8-41

**Published:** 2010-06-30

**Authors:** H Teede, A Deeks, L Moran

**Affiliations:** 1 Jean Hailes Clinical Research Unit, School of Public Health and Preventive Medicine, Monash University, Clayton, Australia; 2 Diabetes Unit, Southern Health, Clayton, Australia

## Abstract

Polycystic ovary syndrome (PCOS) is of clinical and public health importance as it is very common, affecting up to one in five women of reproductive age. It has significant and diverse clinical implications including reproductive (infertility, hyperandrogenism, hirsutism), metabolic (insulin resistance, impaired glucose tolerance, type 2 diabetes mellitus, adverse cardiovascular risk profiles) and psychological features (increased anxiety, depression and worsened quality of life). Polycystic ovary syndrome is a heterogeneous condition and, as such, clinical and research agendas are broad and involve many disciplines. The phenotype varies widely depending on life stage, genotype, ethnicity and environmental factors including lifestyle and bodyweight. Importantly, PCOS has unique interactions with the ever increasing obesity prevalence worldwide as obesity-induced insulin resistance significantly exacerbates all the features of PCOS. Furthermore, it has clinical implications across the lifespan and is relevant to related family members with an increased risk for metabolic conditions reported in first-degree relatives. Therapy should focus on both the short and long-term reproductive, metabolic and psychological features. Given the aetiological role of insulin resistance and the impact of obesity on both hyperinsulinaemia and hyperandrogenism, multidisciplinary lifestyle improvement aimed at normalising insulin resistance, improving androgen status and aiding weight management is recognised as a crucial initial treatment strategy. Modest weight loss of 5% to 10% of initial body weight has been demonstrated to improve many of the features of PCOS. Management should focus on support, education, addressing psychological factors and strongly emphasising healthy lifestyle with targeted medical therapy as required. Monitoring and management of long-term metabolic complications is also an important part of routine clinical care. Comprehensive evidence-based guidelines are needed to aid early diagnosis, appropriate investigation, regular screening and treatment of this common condition. Whilst reproductive features of PCOS are well recognised and are covered here, this review focuses primarily on the less appreciated cardiometabolic and psychological features of PCOS.

## Introduction

Polycystic ovary syndrome (PCOS) is a frustrating experience for women, often complex for managing clinicians and is a scientific challenge for researchers. As research in PCOS is rapidly advancing, it is vital that research evidence is translated to knowledge and action among women, healthcare professionals and policy makers. PCOS is the most common endocrine abnormality in reproductive-age women. The prevalence of PCOS is traditionally estimated at 4% to 8% from studies performed in Greece, Spain and the USA [1-4]. The prevalence of PCOS has increased with the use of different diagnostic criteria and has recently been shown to be 18% (17.8 ± 2.8%) in the first community-based prevalence study based on current Rotterdam diagnostic criteria [5]. Importantly, 70% of women in this recent study were undiagnosed [5]. While the upper limit of prevalence for this study was imputed using estimates of polycystic ovaries (PCO) for women who had not had an ultrasound, non-imputed prevalences were calculated as 11.9 ± 2.4% [5]. PCOS has also been noted to affect 28% of unselected obese and 5% of lean women [5-8]. In 2006, based on US data and traditionally lower prevalence estimates the anticipated economic burden of PCOS in Australia was AU$400 million (menstrual dysfunction 31%, infertility 12% and PCOS-associated diabetes 40% of total costs), representing a major health and economic burden [8]. With regards to fertility, the estimated cost per birth in overweight Australian women with PCOS is high [9]. Promisingly, lifestyle intervention comprising dietary, exercise and behavioural therapy improves fertility and reduces costs per birth significantly [9].

### Aetiology: insulin resistance and hyperandrogenism

**Figure 1:**
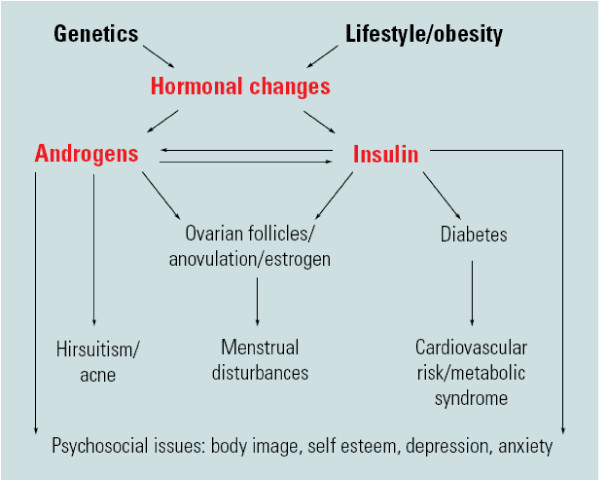
**Schema of aetiology and clinical features including reproductive, metabolic and psychosocial features of polycystic ovary syndrome (PCOS)**. Reproduced with permission from [82].

The exact pathophysiology of PCOS is complex and remains largely unclear. Although a detailed discussion is beyond the scope of this review, the underlying hormonal imbalance created by a combination of increased androgens and/or insulin underpin PCOS (Figure 1). Genetic and environmental contributors to hormonal disturbances combine with other factors, including obesity, ovarian dysfunction and hypothalamic pituitary abnormalities to contribute to the aetiology of PCOS [10,11]. However, greater understanding of pathophysiological contributors in PCOS have been hampered by a lack of ideal methods to assess either hyperandrogenism or insulin resistance. Hyperandrogenism is a well established contributor to PCOS aetiology, detected in around 60% to 80% of cases. Insulin resistance is a pathophysiological contributor in around 50% to 80% of women with PCOS [12], especially in those with more severe PCOS diagnosed on National Institutes of Health (NIH) criteria and in women who are overweight. Conversely, lean women [13] and women with milder PCOS diagnosed on newer European Society for Human Reproduction (ESHRE)/American Society of Reproductive Medicine (ASRM) criteria [14] appear to have less severe hyperinsulinaemia and insulin resistance. , Insulin resistance contributes to metabolic features but also to reproductive features [15] through augmenting androgen production and increasing free androgens by reducing sex hormone binding globulin (SHBG). In this setting of unclear aetiology and mechanisms of insulin resistance, further research is clearly needed.

### Impact of obesity on polycystic ovary syndrome

Obesity and excess weight are major chronic diseases in Western world countries. Obesity increases hyperandrogenism, hirsutism, infertility and pregnancy complications both independently and by exacerbating PCOS [16,17]. In general populations, obesity and insulin resistance further increase type 2 diabetes (DM2) and cardiovascular disease (CVD). Likewise, in PCOS obesity worsens insulin resistance and exacerbates reproductive and metabolic features [16,17]. Furthermore, women with PCOS have increased risk factors for DM2 and CVD, increased impaired glucose tolerance (IGT), DM2 and potentially increased CVD [18]. As obesity rates rise, the public health significance of PCOS will increase [18]. Treatment of obesity through lifestyle intervention is a key treatment strategy in PCOS and improves insulin resistance, reproductive and metabolic features [19].

### Diagnosis of PCOS

Until recently no universally accepted clinical definition existed for PCOS. Over the past three decades, research has highlighted that PCOS is a heterogeneous condition. Symptoms and signs related to PCOS have been evaluated and the initial NIH diagnostic criteria based on oligomenorrhoea/amenorrhoea and clinical or biochemical hyperandrogenism have been broadened in the 2003 Rotterdam or ESHRE/ASRM criteria to include PCO at ultrasound in the key diagnostic criteria [20]. A total of 25% of young women have PCO on ultrasound and the inclusion of PCO in diagnostic criteria has increased the prevalence of PCOS. Recent data indicates that the prevalence of PCOS may be doubled on use of the ESHRE/ASRM criteria with a prevalence of 12% (not imputing presence of polycystic ovaries) to 18% (imputing presence of polycystic ovaries) reported in a community sample [5]. In 2006 the Androgen Excess PCOS Society suggested further modification of the diagnostic criteria to exclude those without symptoms (PCO on ultrasound and oligomenorrhoea/amenorrhoea but no hyperandrogenism) (Table 1) [21]. It should be noted that PCOS is a diagnosis of exclusion and conditions including thyroid dysfunction and hyperprolactinaemia should be excluded biochemically, whilst more rare conditions should be excluded clinically (Cushing's syndrome, virilising tumours, and so on). However, cardiometabolic features and insulin resistance are not currently part of the PCOS diagnostic criteria. This is in part attributable to the lack of accurate methods to measure insulin resistance with measurement not currently recommended in clinical practice [22].

**Table 1:** The different diagnostic criteria for polycystic ovary syndrome (PCOS)

**National Institutes of Health criteria consensus statement **[83]	**European Society for Human Reproduction and Embryology/American Society for Reproductive Medicine consensus statement **[20]	**Androgen Excess Society position statement **[21]
Oligo-ovulation and clinical and/or biochemical signs of hyperandrogenism, and exclusion of other aetiologies*	Two out of three of: oligo-ovulation and/or anovulation, clinical and/or biochemical signs of hyperandrogenism, or polycystic ovaries, and exclusion of other aetiologies*	Hyperandrogenism (hirsutism and/or hyperandrogeniaemia), ovarian dysfunction (oligoanovulation and/or polycystic ovaries), and exclusion of other androgen excess related disorders*

Table adapted from [14], with permission of Oxford University Press, Oxford, UK.

*Congenital adrenal hyperplasia, androgen-secreting tumours, Cushing's syndrome, 21-hydroxylase-deficient non-classic adrenal hyperplasia, androgenic/anabolic drug use or abuse, syndromes of severe insulin resistance, thyroid dysfunction, hyperprolactinaemia.

With the four key diagnostic features, (oligomenorrhoea/amenorrhoea, clinical or biochemical hyperandrogenism and PCO on ultrasound) there are many potential phenotypes (Table 1) [21]. This heterogeneity of the condition is further exacerbated by degree of obesity, insulin resistance, ethnicity and other factors [21]. Both the heterogeneity of PCOS and the lack of an understanding of its aetiology contribute to the evolving diagnostic criteria and ongoing controversy. Currently the ESHRE/ASRM or Rotterdam criteria are the agreed international diagnostic criteria for PCOS, although further research is needed.

### Clinical features of PCOS

Women with PCOS may therefore present with a variety of serious clinical sequalae including psychological problems (reduced quality of life, poor self-esteem, depression, anxiety) [23,24], reproductive manifestations (hirsutism, infertility and pregnancy complications) [25], and metabolic implications (insulin resistance, metabolic syndrome, IGT, DM2 and potentially CVD) [14,26,27] (Figure 1 and Appendix 1). Given the heterogeneous nature of PCOS (Table 1) and the spectrum of clinical features, presentation can vary across the life cycle. PCOS is a chronic condition with psychological and reproductive manifestations usually beginning in adolescence then transitioning to include infertility and increasing metabolic complications over time. However, when combined with obesity, metabolic implications of PCOS such as IGT, DM2 and the metabolic syndrome can present in adolescence [28,29].

## Reproductive features of PCOS

### Ovarian dysfunction and infertility

Ovarian dysfunction usually manifests as oligomenorrhoea/amenorrhoea resulting from chronic oligo-ovulation/anovulation [30]. However, prolonged anovulation can lead to dysfunctional uterine bleeding which may mimic more regular menstrual cycles. The majority of PCOS patients have ovarian dysfunction, with 70% to 80% of women with PCOS presenting with oligomenorrhoea or amenorrhoea. Among those with oligomenorrhoea, 80% to 90% will be diagnosed with PCOS [30]. Among those with amenorrhoea, only 40% will be diagnosed with PCOS as hypothalamic dysfunction is a more common cause [31]. Oligomenorrhoea occurs usually in adolescence, with onset later in life often associated with weight gain. Menstrual irregularity is then often masked by the oral contraceptive pill (OCP), until cessation, when the underlying irregular cycles recur. Menorrhagia can occur with unopposed oestrogen and endometrial hyperplasia, further exacerbated by elevated oestrogen levels in obesity. Whilst inadequate research exists, it is generally recommended that greater than four cycles per year may protect the endometrium. Women with regular menstrual cycles can also now be diagnosed with PCOS based on newer diagnostic criteria (Table 1) [21].

PCOS is the most common cause of anovulatory infertility. It accounts for 90% to 95% of women attending infertility clinics with anovulation. However 60% of women with PCOS are fertile (defined as the ability to conceive within 12 months), although time to conceive is often increased [30]. In those with PCOS and infertility, 90% are overweight. Obesity independently exacerbates infertility, reduces efficacy of infertility treatment and induces a greater risk of miscarriage [30]. There is currently an active debate about the appropriate limit for body mass index for assisted reproduction therapies, given the reduced success rates and the demonstrated risks of pregnancy in overweight women [32]. Ideally, weight should be optimised prior to pregnancy. Age-related infertility also exacerbates infertility and timely planning of families may warrant discussion.

### Hyperandrogenism

The clinical and/or biochemical signs of androgen excess in PCOS result from increased synthesis and release of ovarian androgens. Elevated luteinising hormone and insulin synergistically increase androgen production. Insulin resistance leads to hyperinsulinaemia, reduces SHBG and raises free circulating testosterone and together, hyperandrogenism and hyperinsulinaemia impairs ovarian follicle development. Clinical hyperandrogenism primarily includes hirsutism, acne and male pattern alopecia [21]. Hirsutism is defined in females as male type terminal hair growth and distribution [33]. PCOS is a common cause of hirsutism occurring in approximately 60% of cases, however this varies with race and degree of obesity [21]. Hirsutism should be assessed with a standardised scoring system (Ferriman-Gallwey score). Acne affects one third of cases and is not particularly specific for PCOS [33]. Male pattern hair loss (androgenic alopecia) is less frequently seen in PCOS cases, as it generally requires a familial predisposition. Other features of hyperandrogenism include virilisation, which, especially if presenting with clitoromegaly and rapid onset, requires exclusion of other causes including adrenal or ovarian androgen-secreting tumours.

Biochemical hyperandrogenism is present in most patients with PCOS. Measurement of biochemical androgens in PCOS is limited by poor accuracy and reproducibility of assays, which are designed for significantly higher male androgen levels. Free androgen index measurements are generally recommended, derived in the lab from SHBG and total testosterone measurements [33]. Dehydroepiandrosterone sulfate (DHEAS) and androstenedione are not routinely recommended in PCOS [21].

## Metabolic features of PCOS

### Dyslipidaemia

Dyslipidaemia is common in PCOS compared to weight matched controls [34-37], with higher triglycerides and lower high density lipoprotein cholesterol [35]. The dyslipidaemia occurs independent of body mass index (BMI) [35,38] , however there is a synergistic deleterious effect of obesity and insulin resistance in PCOS analogous to that seen in DM2. The causes of dyslipidaemia in PCOS are again multifactorial. Insulin resistance appears to have a pivotal role mediated in part by stimulation of lipolysis and altered expression of lipoprotein lipase and hepatic lipase [35].

### Insulin resistance and abnormal glucose metabolism

Insulin resistance occurs in around 50% to 80% of women with PCOS [12], primarily in the more severe NIH diagnosed PCOS and in those who are overweight. Lean women [13] and milder Rotterdam diagnosed PCOS [14] appear to have less severe insulin resistance. A full discussion of the complex mechanisms involved in insulin resistance, hyperinsulinaemia, DM2 and CVD is beyond the scope of this review. Mechanisms involved in insulin resistance are likely to be complex with genetic and environmental contributors. Specific abnormalities of insulin metabolism identified in PCOS include reductions in secretion [39,40], reduced hepatic extraction [40], impaired suppression of hepatic gluconeogenesis [41] and abnormalities in insulin receptor signalling [42]. Interestingly, there is a paradoxical expression of insulin resistance in PCOS whereby insulin-stimulated androgen production persists while its role in glucose metabolism is impaired [42]. Therefore, insulin resistance in PCOS results in hyperinsulinaemia with its associated diverse and complex effects on regulating lipid metabolism, protein synthesis and modulation of androgen production. The cause of insulin resistance is likewise complex and multifactorial with genetic and environmental contributors [15]. Lean women with PCOS often but not always [13] have abnormalities of insulin secretion and action compared to weight-matched control subjects [41]. Where a woman with PCOS is overweight, she may also demonstrate extrinsic insulin resistance associated with adiposity, which is potentially mechanistically distinct from the insulin resistance present in lean women with PCOS. In women with insulin resistance and PCOS, only a subgroup develop coexistent pancreatic insufficiency with β cell failure and go on to DM2. In this setting, insulin output cannot overcome resistance and hyperglycaemia develops. Women with PCOS are at increased risk of developing IGT and DM2 with prevalence rates of 31.3% and 7.5%, respectively, compared to 14% for IGT and 0% for DM2 in age-matched and weight-matched non-PCOS control women [27].

Women with PCOS also develop abnormal glucose metabolism at a younger age and may demonstrate a more rapid conversion from IGT to DM2 [43]. The rate of conversion from IGT to DM2 in a general Australian population was estimated in the large cohort Australian Diabetes, Obesity and Lifestyle (AusDiab) study at 2.9% per year for young females [44]. Another Australian study has reported a substantially higher conversion rate (8.7% per year over 6.2 years) in women with PCOS [45], however this has not been uniformly reported [46]. Women with PCOS also have higher gestational diabetes (GDM) risk, with a recent meta-analysis reporting an odds ratio (OR) of 2.94 [25]. The risk of GDM occurs both independent of and is exacerbated by obesity [27,47]. Whilst there are few adequately powered studies assessing natural history of IGT, DM2 and CVD in PCOS and there is a need for further research, the International Diabetes Federation has identified PCOS as a significant non-modifiable risk factor associated with DM2 [48].

It is increasingly clear that IGT is also a clinically relevant state where early identification and intervention improve long-term outcomes [49]. IGT has been found to increase the risk of CVD, mortality and progression to DM2 in general populations [44]. Recent population-based data noted a mortality rate of 5.5% over 5 years for those with IGT versus 1.9% with normal glucose tolerance [44]. Furthermore, lifestyle intervention, metformin and glitazones can prevent IGT progression to DM2 [49], strengthening the argument for early detection of IGT, including in high-risk PCOS women

There are currently no generic guidelines for IGT screening, only for DM2 based on fasting glucose or more recently on HbA1c as a first line. However, impaired fasting glucose is a poor predictor of IGT in women in general [50] and in PCOS [27,43]. Hence the ESHRE/ASRM-sponsored PCOS Consensus Workshop Group recommend an oral glucose tolerance test in all overweight women with PCOS [51]. Furthermore, emerging data shows increased risk of metabolic complications in first-degree family members of women with PCOS [52-56]. Screening for metabolic conditions may be also warranted in relatives of women with PCOS, although this requires further research.

### Cardiovascular disease risk

Alongside insulin resistance, metabolic syndrome, IGT and DM2, women with PCOS also have increased novel cardiovascular risk factors (inflammation, oxidative stress and impaired fibrinolysis) [14]. Also, increased early clinical and subclinical markers of atherosclerosis seen in PCOS (endothelial dysfunction, impaired pulse wave velocity, increased carotid intima media wall thickness, presence of carotid plaque and increased coronary artery calcification) [34,57] are further exacerbated by obesity [27,58,59]. Given that large longitudinal cohort studies have reported up to 65% of CVD deaths occur in subjects with impaired glucose metabolism [60] and that IGT and DM2 are increased in PCOS, it would be expected that women with PCOS would have increased CVD risk. There is currently a lack of long-term studies in PCOS to appropriately address CVD risk. Some studies support an increased risk of CVD in PCOS [18], but these findings are not universal [61] and further research is needed. A recent study in postmenopausal women with premenopausal features of PCOS noted higher prevalence of angiographic coronary artery disease and that PCOS was associated with worsened cardiovascular event-free survival [18].

### Psychological features of PCOS

Most research has focused on the biological and physiological aspects of the syndrome. The challenges to feminine identity and body image due to obesity, acne and excess hair, as well infertility and long-term health-related concerns compromise quality of life and adversely impact on mood and psychological well-being [23,62]. Limited studies to date have reported that women who have PCOS are more prone to depression, anxiety, low self-esteem, negative body image, and psychosexual dysfunction [63,64]. The other critical aspect of psychosocial impact in PCOS is the negative impact of mood disturbance, poor self-esteem and reduced psychological well-being on motivation and on ability to implement and sustain successful lifestyle changes that are critical in this condition [19]. These issues all need to be explored and addressed as part of PCOS assessment and management.

### Investigations and assessment in PCOS

There is no single diagnostic test for PCOS. Key investigations include prolactin and thyroid stimulating hormone to exclude other disorders and testosterone, SHBG and free androgen index to assess androgen status [33]. Other investigations include a pelvic ultrasound for ovarian morphology and endometrial thickness. An oral glucose tolerance test (rather than fasting glucose) and lipid profiles are appropriate in all women at diagnosis and 1 to 2 yearly after this, where women are overweight or have an increased risk of DM2 (for example, family history of DM2 in first-degree relatives, increased age or high-risk ethnic group). As noted, insulin levels should not be measured in clinical practice because of assay variability and inaccuracy. Metabolic syndrome and abnormal glucose metabolism best reflect insulin resistance in this population.

## Treatment of PCOS

### Targeted approach to therapy

**Figure 2:**
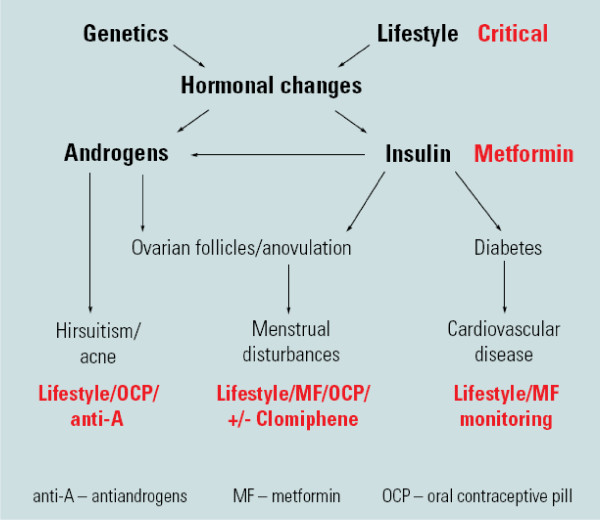
**Summary of a targeted approach to therapy in polycystic ovary syndrome (PCOS)**. Reproduced with permission from [82].

Treatment options need to be tailored to the clinical presentation. Education on short-term and long-term sequalae of PCOS from a reliable independent source is important in allaying anxiety and minimising the impact of illness in chronic disease (Table 2). As a prelude to treatment psychological features need to be acknowledged, discussed and counselling considered [65], to enable lifestyle change which is unlikely to be successful without first addressing education and psychosocial issues (Figure 2 and Appendix 2).

**Table 2:** Evidence-based government funded resources to inform consumers and/or health professionals in polycystic ovary syndrome (PCOS)

Resource	Description
http://www.managingpcos.org.au	Evidence-based independent consumer and health professional information

http://www.jeanhailes.org.au	Evidence-based independent consumer and health professional information

PCOS patient fact sheets	Freely available: link from website above

### Weight loss, exercise and lifestyle interventions

Lifestyle change is first line treatment in an evidence-based approach in the management of the majority of PCOS women who are overweight [19]. Furthermore, prevention of excess weight gain should be emphasised in all women with PCOS of both normal or increased body weight. As little as 5% to 10% weight loss has significant clinical benefits improving psychological outcomes [66], reproductive features (menstrual cyclicity, ovulation and fertility) [9,67] and metabolic features (insulin resistance and risk factors for CVD and DM2). Evidence shows that lifestyle change with small achievable goals results in clinical benefits even when women remain in the overweight or obese range, [9,68,69]. Standard dietary management of obesity and related comorbidities [70] is a nutritionally adequate, low fat (approximately 30% of energy, saturated fat approximately 10%), moderate protein (approximately 15%) and high carbohydrate intake (approximately 55%), with increased fibre-rich wholegrain breads, cereals, fruits and vegetables and moderate regular exercise. A moderate energy reduction diet (500 to 1,000 kcal/day reduction) reduces body weight by 7% to 10% over a period of 6 to 12 months. Simple and practical tips that can be covered in minutes in medical consultation include targeting fruit juice, soft drinks, portion sizes and high-fat foods. Incorporating simple moderate physical activity including structured exercise (at least 30 min/day) and incidental exercise increases weight loss and improves clinical outcomes in PCOS, compared to diet alone [71]. Exercise alone also improves clinical outcomes. As in the general population, goals for exercise must focus on overall health benefits not weight loss *per se*.

Fad diets are not encouraged as short-term weight loss, if achieved, is rarely sustainable [72]. The advantages of specific dietary approaches over that of caloric restriction alone are still unclear and more research is needed. Proposed specific dietary approaches in PCOS include high protein, low carbohydrate and low glycaemic index/glycaemic load diets. A number of small studies assessing specific dietary approaches in PCOS show similar results for diets moderately increased in dietary protein or carbohydrate [73-75] with one study reporting greater weight loss where a high protein supplement was added to a standard energy reduced diet [76]. Two small studies have assessed very low carbohydrate diets in PCOS, and one study reported on an audit of reduced glycaemic load diets in clinical practice. While reductions in weight, BMI, waist circumference, fasting insulin or testosterone were reported, these studies lacked a control group [77-79]. The current evidence suggests that a range of dietary strategies, as long as they are safe, nutritionally adequate and sustainable in the long term, will similarly improve weight, and reproductive and metabolic features in PCOS [19].

### Pharmacological therapy in PCOS

There is currently no ideal medical PCOS therapy that fully reverses underlying hormonal disturbances and treats all clinical features. The OCP does improve hyperandrogenism and insulin sensitisers (primarily Metformin) reduce insulin resistance in PCOS [80]. Generally, medical therapy is targeted to symptoms and should not be used as an alternative to lifestyle therapy in PCOS (Figure 2). Simple medical therapy is summarised in Appendix 2. The OCP has long been used in PCOS to induce regular cycles, protect the endometrium and treat hyperandrogenism. Mechanisms of action include a significant first pass hepatic effect, increasing production of hepatic proteins including sex hormone binding globulin. This reduces free circulating androgen levels, even with low dose OCPs. This important mechanism of antiandrogenic action does not occur with progestin alone or non-oral oestrogen containing contraceptive preparations. The OCP also reduces ovarian androgen production. There has been concerning data that the OCP can increase insulin resistance and worsen glucose tolerance. Studies are inadequate and data conflicting with more research needed, however consideration should be given to cardiometabolic effects of medical therapy and low dose OCP preparations may be a preferable alternative, with similar efficacy and reduced cardiometabolic effects [80].

Metformin has had an increasing role in PCOS management [22,81], improving clinical features (ovulation, cycle regulation, and potentially hirsutism) with positive cardiometabolic effects [22,81]. It does not appear to induce weight loss, although based on studies in DM2 it may assist in preventing future weight gain. Based on International Diabetes Federation recommendations [48,80], metformin has a role in prevention of diabetes where lifestyle therapy is inadequate. Given the increased insulin resistance and high risk of DM2, this includes PCOS especially if other risk factors including excess weight, family history of DM2, metabolic syndrome or prediabetes exist [22]. In infertility, the role of metformin remains controversial. It does reduce hyperstimulation in those on other fertility therapies, however more research here is important. When using metformin it is better tolerated if started at 500 mg of slow release daily and increased over weeks to months to reach 2 g daily. Lactic acidosis is a rare side effect in those with other significant illnesses including renal impairment [22]. It is important to note that neither metformin nor the OCP are approved by most regulatory authorities specifically for PCOS. The OCP is indicated for contraception and metformin for the treatment of diabetes. However, both treatments are recommended by international and national endocrine societies and are evidence based [20]. A detailed discussion of infertility therapy is beyond the scope of this review, however clomiphene is generally used as initial medical therapy.

## Conclusions

PCOS is a common complex condition in women associated with psychological, reproductive and metabolic features. It is a chronic disease with manifestations across the lifespan and represents a major health and economic burden. Both hyperandrogenism and insulin resistance contribute to pathophysiology of PCOS. Insulin resistance occurs in the majority of women with PCOS, especially those who are overweight, and these women have a high risk of metabolic syndrome, prediabetes and DM2. Management should focus on support, education, addressing psychological factors and strongly emphasising healthy lifestyle with targeted medical therapy as required. Treatment for the large majority is lifestyle focused and an aggressive lifestyle-based multidisciplinary approach is optimal in most cases to manage the features of PCOS and prevent long-term complications. Small achievable goals of 5% loss of body weight result in significant clinical improvement even if women remain clinically in the unhealthy overweight or obese range. Addressing hyperandrogenism is clinically important and monitoring for and managing longer-term metabolic complications, including dyslipidaemia, IGT, DM2, and cardiovascular risk factors, is crucial. Consideration should be given to screening high-risk family members for metabolic abnormalities also. Overall, further research is needed in this complex condition. In the interim, comprehensive evidence-based guidelines are needed to guide consumers and clinicians in optimal PCOS management.

## Competing interests

The authors declare that they have no competing interests.

## Authors' contributions

HT, AD and LM all made substantial contributions to conception and design of the paper, were involved in drafting the manuscript and revising it critically for important intellectual content and have given final approval of the version to be published.

## Appendix 1

### Reproductive, metabolic and psychosocial features of polycystic ovary syndrome (PCOS)

#### Clinical features of PCOS

(1) Reproductive features: hyperandrogenism, hirsutism, ovulatory and menstrual dysfunction, infertility, complications in pregnancy, miscarriage, pregnancy-induced diabetes (gestational diabetes), pregnancy-induced hypertensive disorders and neonatal complications and increased endometrial hyperplasia.

(2) Metabolic features: insulin resistance, metabolic syndrome, dyslipidaemia, high rates of premature impaired glucose tolerance, type 2 diabetes and increased cardiovascular risk factors.

(3) Psychological features: anxiety, depression, poor self-esteem, reduced quality of life, negative body image.

## Appendix 2

### Summary of treatment options in polycystic ovary syndrome (PCOS)

#### Oligomenorrhoea/amenorrhoea

• Lifestyle change (5% to 10% weight loss and structured exercise).

• Oral contraceptive pill (OCP; low oestrogen doses, for example 20 μg may be preferable).

• Cyclic progestins (for example, 10 mg medroxyprogesterone acetate for 14 days every 2 to 3 months).

• Metformin (improves ovulation and menstrual cyclicity).

#### Hirsutism treatment recommendations

• Cosmetic therapy.

• Laser treatment.

• Eflornithine cream can be added and may induce a more rapid response.

#### Pharmacological therapy

• Medical therapy if patient concerned about hirsutism and cosmetic therapy ineffective, inaccessible or unaffordable.

• Primary therapy is the OCP (monitor glucose tolerance in those at risk of diabetes).

• Antiandrogen monotherapy should not be used without adequate contraception.

• Trial therapies for ≥ 6 months before changing dose or medication.

• Combination therapy: if ≥ 6 months of OCP is ineffective, add antiandrogen to OCP (daily spironolactone 50 mg twice a day or cyproterone acetate 25 mg/day for days 1 to 10 of the active OCP tablets).

#### Infertility

• Obesity independently exacerbates infertility and reduces effectiveness of interventions. Maternal and foetal pregnancy risks are greater and long-term metabolic outcomes in the child are related to maternal weight at conception. Consistent with international guidelines, women who are overweight prior to conception should be advised on folate, smoking cessation, weight loss and optimal exercise, prior to additional interventions.

• Given age-related infertility, advise women to optimise family planning.

• Infertility therapies may include clomiphene, gonadotrophins and *in vitro *fertilisation.

#### Metabolic syndrome, prediabetes, diabetes and cardiovascular disease risk

Obesity independently causes metabolic complications; lifestyle/exercise is critical:

• Lifestyle change with a 5% weight loss reduces diabetes risk by approximately 50% to 60% in high-risk groups [49].

• Metformin* reduces the risk of diabetes by approximately 50% in high-risk groups [49].

*Metformin and the OCP are not currently approved for use to manage PCOS by many regulatory bodies. The OCP is primarily indicated for contraception and metformin for diabetes. However, their use is recommended by international and national specialist societies and is evidence based [22].

## Pre-publication history

The pre-publication history for this paper can be accessed here:

http://www.biomedcentral.com/1741-7015/8/41/prepub
